# Nucleo-mitochondrial asymmetry profiles the proliferative engine and spatial niche reconstruction in clear cell renal cell carcinoma

**DOI:** 10.3389/fimmu.2026.1838182

**Published:** 2026-06-12

**Authors:** Shansen Peng, Zhouzhou Xie, Ting Hu, Xia Li, Chunmei Yan, Chuyang Jiang, Huiming Jiang, Guihao Zhang, Nanhui Chen

**Affiliations:** 1 Affiliated Meizhou Hospital of Shantou University Medical College, Meizhou, China; 2 Department of Urology, Meizhou People’s Hospital (Meizhou Academy of Medical Sciences), Meizhou, China

**Keywords:** clear cell renal cell carcinoma, MT-CO1, nucleo- mitochondrial asymmetry, scRNA-seq, stRNA-seq

## Abstract

**Background:**

Tumor heterogeneity is the key driver of disease progression and therapeutic resistance in clear cell renal cell carcinoma (ccRCC). Within this landscape, mitochondrial (MT) heterogeneity has emerged as a critical but poorly understood feature. This study identified a specific manifestation of MT heterogeneity termed “nucleo-mitochondrial expression asymmetry (NMA)”. It is characterized by a dysregulated burst of mitochondrial DNA (mtDNA)-encoded genes compared to the nuclear genome, marking a pivotal tipping point in tumor proliferation and spatial reconstruction.

**Methods:**

We employed an integrative multi-omics approach combining single-cell RNA sequencing (scRNA-seq), spatial transcriptomics (stRNA-seq), and mass spectrometry imaging (MSI)-based spatial metabolomics from the Tongji Renal Cell Carcinoma (TJ-RCC) cohort. To identify and characterize the profound NMA malignant subpopulations, we utilized Gaussian Mixture Model (GMM) clustering, CytoTRACE 2 for differentiation potential, and scFEA for metabolic flux inference. We implemented neighborhood and pseudo-spatiotemporal map (pSM) analyses to quantify spatial reconstruction. We validated these findings through mitochondrially encoded cytochrome *c* oxidase I (MT-CO1) immunohistochemistry (IHC) in an independent cohort of 53 patients.

**Results:**

We identified a unique malignant subpopulation (C0) defined by NMA, where nucleo-mitochondrial coordination significantly decreased to R = 0.30 compared to R = 0.50 in other clusters. C0 functioned as a proliferative engine, exhibiting the highest ribosomal activity, peak differentiation potential, and concentrated G2M/S-phase activity. Metabolic modeling and MSI revealed that C0-dominant regions act as metabolic hubs, correlating with total metabolic flux (R = 0.631) and the physical accumulation of tricarboxylic acid (TCA) cycle intermediates. Spatially, C0 abundance was highly predictive of global MT gene scores (R = 0.852). As ccRCC progressed, NMA-driven niches underwent a dramatic reconstruction: transitioning from an “immune-active core” in the early stages to stroma-shielded “metabolic islands” in the advanced stages. Furthermore, we observed a resurgence of NMA and the C0 subpopulation in metastatic lesions. Clinical validation confirmed that MT-CO1 protein levels—a histological proxy for NMA—positively correlated with the proliferation marker Ki67 (r = 0.702) and served as an independent prognostic factor for overall survival.

**Conclusion:**

This study characterized NMA as a hallmark of ccRCC progression and spatial niche reconstruction, offering a novel, clinically actionable framework for metabolic risk stratification via MT-CO1.

## Graphical Abstract

**Figure:**
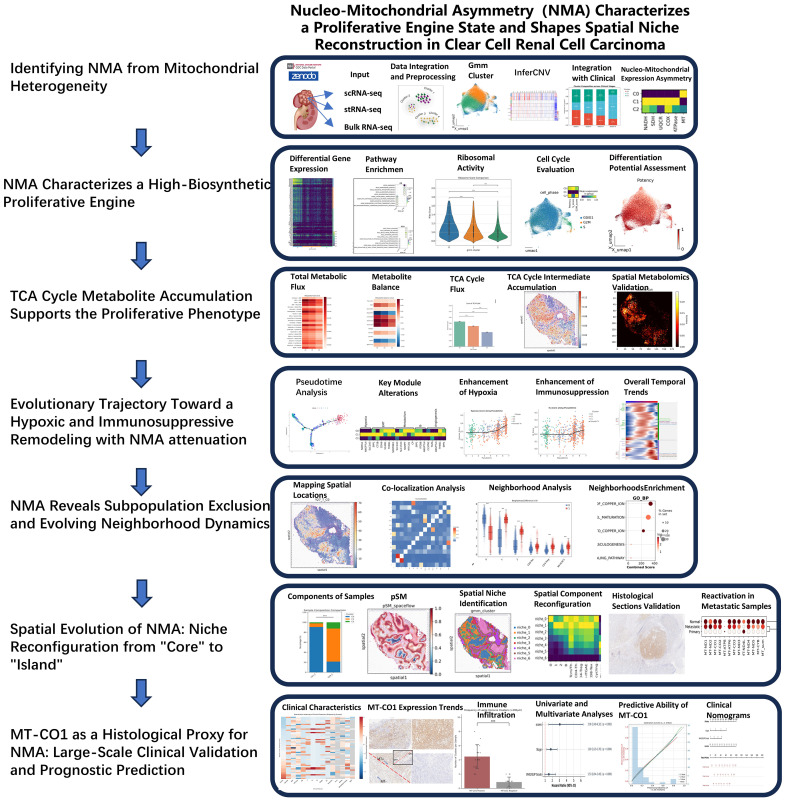

## 1 Introduction

Renal cell carcinoma (RCC) is a globally prevalent malignancy, with approximately 430,000 new cases diagnosed annually (1). Clear cell renal cell carcinoma (ccRCC) is the primary histological subtype of RCC, accounting for nearly 70% of all cases (2). Disease progression and therapeutic resistance in ccRCC are largely driven by tumor heterogeneity (3). Within tumor heterogeneity, mitochondrial (MT) heterogeneity has emerged as a pivotal but still not fully clarified feature.

Historically, ccRCC was characterized as a predominantly glycolytic tumor, a hallmark determined by the core VHL–HIF pathway (4–6). The conventional paradigm held that VHL–HIF pathway activation systematically suppresses MT function, leading to MT downregulation. This suppression is mediated by significant reductions in MT copy number variations (CNVs), inhibited oxidative phosphorylation (OXPHOS), and the redirection of glutamine toward reductive carboxylation (7, 8). However, this monolithic view has been challenged by emerging evidence of significant MT expression diversity within the tumor mass. Recent single-cell studies have identified ccRCC subpopulations with high MT gene expression (9, 10). This suggests that some malignant cells may bypass traditional VHL–HIF metabolic constraints, driving transformation via energy stress, epigenetic remodeling, and other mechanisms to drive MT heterogeneity (11).

Building on this, a 2019 study demonstrated that SETD2 loss can induce a switch toward enhanced aerobic respiration efficiency (12). Furthermore, a 2024 *Nature* study revealed that mitochondrial-encoded respiratory complex I can be overexpressed in specific genetic backgrounds to directly promote metastasis (13). Despite these observations of elevated MT activity, the coordination and influence between MT and nuclear genomes remain unclarified. Specifically, we believe that there exists a phenomenon called “nucleo-mitochondrial expression asymmetry (NMA)”. It is characterized by a dysregulated burst of mitochondrial DNA (mtDNA)-encoded genes relative to the stable nuclear genome. This asymmetry may represent a critical tipping point in ccRCC evolution.

Recent evidence indicates that some malignant clones can gain higher proliferative potential through enhanced mitochondrial biosynthesis. Furthermore, these clones possess the capacity to remodel the spatial environment, thereby bolstering therapeutic resistance and facilitating metastatic progression (14, 15). Against this backdrop, investigating the biological properties linked by NMA in ccRCC and how these niches are reconstructed is essential to uncovering the physical principles of ccRCC progression. The MT genome encodes a total of 13 proteins and is transcribed as a polycistronic unit, which implies high collinearity among these 13 MT proteins (16, 17). Mitochondrially encoded cytochrome *c* oxidase I (MT-CO1) serves as a core catalytic subunit of complex IV. In this role, it acts as a key determinant of cellular bioenergetic capacity (18, 19). Therefore, the expression level of MT-CO1 provides a robust histological proxy for global MT transcriptional activity, further quantifying the clinical application value of NMA.

In this study, we employed an integrative multi-omics approach to investigate MT heterogeneity in primary ccRCC. This strategy combined single-cell RNA sequencing (scRNA-seq), spatial transcriptomics (stRNA-seq), and mass spectrometry imaging (MSI)-based spatial metabolomics. We identified a unique subpopulation, defined by NMA, that functions as an aggressive “proliferative engine” driving early clonal expansion. By tracking this subpopulation across stages, we revealed a dramatic spatial niche reconstruction: a transition from an immune-active central core to stromal-shielded “metabolic islands”. These findings were substantiated by protein-level validation in an independent cohort of 53 cases. This validation demonstrated that NMA (MT-CO1) provides a stage-specific readout of progression. Our work establishes a novel spatial-metabolic framework that bridges intracellular NMA with macroscopic tumor architecture and clinical prognosis.

## 2 Methods

### 2.1 Multi-omics data acquisition and preprocessing

Multi-omics data for the TJ-RCC cohort were obtained from Zenodo (https://zenodo.org/record/8063124). This dataset included bulk RNA-seq, scRNA-seq, stRNA-seq, and spatial metabolomics from the same samples. The bulk RNA-seq dataset comprised 100 ccRCC samples and 50 adjacent normal tissues, with raw counts normalized using transcripts per million (TPM).

For scRNA-seq analysis, 20 samples from the bulk RNA-seq cohort were selected. Data processing was performed using Python packages “Scanpy” and “omicverse” (20, 21). All samples were categorized as either “tumor” or “adjacent normal”. Since the research focused on MT genes, cells with high MT gene expression were not filtered out. Only cells with fewer than 500 Unique Molecular Identifiers (UMIs) or fewer than 250 detected genes were removed. Gene expression in each cell was normalized to account for sequencing depth differences, followed by log transformation. The top 3,000 highly variable genes were calculated, and scaled principal component analysis was performed, retaining the first 50 principal components. Batch effects were corrected using Harmony, and visualization was achieved using Uniform Manifold Approximation and Projection (UMAP) (22).

The stRNA-seq dataset included 12 cancer tissues sampled from the 20 scRNA-seq specimens. Spots without tissue coverage were removed by comparison with pathological sections, and genes with total expression counts below 100 across all spots were filtered out. A custom normalization procedure was implemented. After deconvolution to determine cellular composition at each spot, the total cell count per spot was calculated, and gene expression data were normalized by dividing by this count. The total gene expression at each spot was standardized to 1,000 to eliminate technical bias. Finally, the normalized data were multiplied by the total cell count to restore the spot’s total expression level.

For spatial metabolomics, m/z values and spatial coordinate information for each spot were extracted. Data for the The Cancer Genome Atlas Kidney Renal Clear Cell Carcinoma (TCGA-KIRC) cohort were obtained from The Cancer Genome Atlas database (https://portal.gdc.cancer.gov/). After removing samples with incomplete datasets, 533 KIRC patients with gene expression data normalized by TPM were included. Immunohistochemical staining slides for MT genes were acquired from the Human Protein Atlas project (https://www.proteinatlas.org/).

### 2.2 Cell annotation and deconvolution

scRNA-seq clusters were annotated based on marker genes from previous literature (5). A two-step annotation strategy was employed, involving initial categorization into major cell types, followed by refinement into subtypes. Annotation accuracy was verified using the automated annotation tool “MetaTiME” (23).

For the deconvolution of stRNA-seq data to determine cell abundance at each spot, the Python package “cell2location” (24) was utilized. Gene expression features from each annotated cell type in the scRNA-seq data were first extracted as a reference. Spatial transcriptomics data were then modeled as a linear combination of different cell type expression features. This approach assumes that gene expression at each spatial location is a weighted sum of cell type expression features. Hyperparameters N_cells_per_location were set to 30 and detection_alpha to 20. Using a Bayesian framework, prior distributions were introduced to constrain cell type proportions and expression features. Cell type proportions at each spatial location were estimated through posterior inference. A sample was drawn from the fifth percentile of the posterior distribution of cell abundance.

### 2.3 Gaussian mixture model clustering

A Gaussian Mixture Model (GMM) is a probability-based unsupervised clustering method (25). It assumes that data are generated from multiple Gaussian distributions, with each distribution corresponding to a cluster. Parameters for each Gaussian distribution and the probability of each data point belonging to each cluster are estimated by maximizing the likelihood function. GMM clustering was applied to all cancer cells in the scRNA-seq data based on the “KEGG_OXIDATIVE_PHOSPHORYLATION” gene set, with the number of clusters set to three. For stRNA-seq, GMM clustering was performed on all spots using the pseudo-spatiotemporal map (pSM) calculated using the Python package “SpaceFlow” to define six to seven spatial niches.

### 2.4 Inferring cancer cell copy number variations

To efficiently calculate CNVs, the Python package “Infercnvpy” was used to infer CNVs from scRNA-seq data (https://github.com/icbi-lab/infercnvpy). This tool identifies copy number changes in genomic regions by comparing gene expression patterns between target cells and reference cells. Normal kidney epithelial cells were designated as reference cells. The sliding window was set to 250, and the dynamic threshold to 1.5.

### 2.5 Ribosomal gene identification

The Python package “cosg”, a cosine similarity-based method for more accurate and scalable marker gene identification (26), was used to identify characteristic ribosomal marker genes in the C0 subpopulation.

### 2.6 Survival analysis

The KaplanMeierFitter and logrank_test modules from the Python package “lifelines” were used to calculate survival differences between samples with high and low MT gene expression (27). Statistical significance was determined using the log-rank test.

### 2.7 Pseudotime analysis

The R package “Monocle 2” was employed to analyze cellular trajectory and gene expression dynamics (28). This method reconstructs cell dynamics during development or differentiation through dimensionality reduction, ordering, and modeling. The pseudotime of each cell in the C0, C1, and C2 subpopulations was analyzed, followed by ordering and analysis based on their pseudotime values.

### 2.8 Assessment of cancer cell differentiation potential

CytoTRACE 2 is a computational method that predicts cell potency categories and absolute developmental potential from scRNA-seq data (29). It classifies cells based on their developmental potential, ranging from pluripotent and multipotent cells with broad differentiation capacity to terminally differentiated phenotypes. We used CytoTRACE 2’s pre-trained model to predict differentiation potential across tumor subgroups. Each cell received a differentiation potential score between 0 and 1, with higher scores indicating greater differentiation potential.

### 2.9 Cell cycle analysis

The “sc.tl.score_genes_cell_cycle” function from the Python package “scanpy” was used to determine cell cycle phases across tumor subpopulations (22). Based on S-phase and G2M-phase gene scores, all tumor cells were classified into the G0/G1, S, or G2M phase.

### 2.10 Identification of key genes associated with cell fate determination

The Python package “CellRank” was used to analyze gene expression trends and identify key genes associated with cell fate determination (30). Pseudotime data generated by “Monocle 2” were utilized as input, and expression changes for all genes during the C0-to-C1 transition were calculated. After model fitting and filtering, the top 50-ranked genes were extracted as key determinants of cell fate.

### 2.11 Cell-pathway co-localization analysis

To explore spatial interactions and co-localization patterns of features such as genes and cell types, the MISTy framework within the Python package “LIANA” was employed (31). In 10X Visium stRNA-seq, MISTy classifies co-localization relationships into three types: Intra, referring to co-localization within a single spot; Juxta, describing co-localization between a central spot and its six immediate neighboring spots; and Para, extending to co-localization between a central spot and its 18 surrounding neighboring spots.

Deconvolution was first applied to estimate the proportion of cell types at each spot. These estimates were then processed using a random forest model to calculate the three types of co-localization relationships between different cell types. Subsequently, the top 1,000 genes most strongly associated with common biological pathways, along with their corresponding weights, were identified. These genes and weights served as input in a linear model to determine the Juxta and Para co-localization relationships between cell types and biological pathways.

### 2.12 Identification of pSM

“SpaceFlow” is a Python package based on deep graph networks for analyzing stRNA-seq data (32). It encodes stRNA-seq into low-dimensional embeddings and integrates cellular spatiotemporal relationships through pSM. This approach helps identify spatial domains with coherent expression patterns. High signals in pSM often indicate core cancer progression areas, while low signals suggest immune infiltration areas. After preprocessing stRNA-seq data, we identified pSM with a spatial regularization intensity of 0.1. Then, we used GMM to calculate spatial niches based on pSM.

### 2.13 Identification of spatial neighborhood differences

In this study, custom code was developed to calculate the cellular and gene neighborhoods of spatial sites. The process was carried out as follows. First, spatial coordinates for each site were extracted from the spatial transcriptomic data. Next, the K-nearest neighbor algorithm was applied to identify the six closest neighbors for each spatial site within the sample. Distances between each site and its neighbors were computed and weighted, followed by the removal of unsuitable neighbors to construct a spatial neighborhood network. Finally, for each spatial site, the cell types and gene expression values of its neighbors were aggregated.

Then, spatial sites primarily composed of the C0 and C1 subpopulations were focused on. For these sites, differential expression analysis [differentially expressed genes (DEGs)] was performed on their cellular and gene neighborhoods. Significant differences in both cellular and gene neighborhoods were identified using the Wilcoxon test.

### 2.14 Enrichment analysis

Gene Ontology enrichment analysis was performed on the top 500 genes with the largest fold changes in gene neighborhoods between the C0 and C1 subpopulations using the Python package “gseapy” (33). Gene set enrichment analysis (GSEA) was also performed using the Python package “gseapy”.

### 2.15 Characterizing metabolic features of spatial regions

The Python package “scFEA” is a computational method that uses graph neural networks to estimate metabolic flux from transcriptomic data (34). It represents the metabolic network as a directed factor graph. In this graph, each metabolic module is a factor node, and each intermediate metabolite is a variable node. The method employs a likelihood function to describe the balance of metabolic fluxes. The input for scFEA includes gene expression data and a representation of the metabolic network. The flux of each module is modeled using a multi-layer fully connected neural network. The loss function of scFEA incorporates three key constraints: 1) flux balance, 2) non-negativity of fluxes, and 3) relative scaling of fluxes. These constraints are weighted by hyperparameters α, β, and γ. In this study, the hyperparameters were set to α = 1, β = 1, and γ = 0.5. These values were chosen to ensure both high predictive accuracy and biological interpretability of the results.

### 2.16 Clinical cohort and tissue samples

To validate the computational findings, an independent validation cohort of 53 patients diagnosed with primary ccRCC was enrolled at Meizhou People’s Hospital. Formalin-fixed paraffin-embedded (FFPE) tissue blocks were obtained from the surgical pathology archives. Clinical staging was determined according to the eighth edition of the American Joint Committee on Cancer TNM staging system (35), and pathological grading was performed using the World Health Organization/International Society of Urological Pathology (WHO/ISUP) system (36). The study was approved by the Institutional Review Board of Meizhou People’s Hospital, and informed consent was obtained from all participating patients.

### 2.17 Immunohistochemistry

Serial sections (4 μm thick) were prepared from the FFPE blocks. Immunohistochemical staining was performed using a standard streptavidin–peroxidase protocol (37). Briefly, sections were deparaffinized in xylene and rehydrated through a series of graded alcohols. Antigen retrieval was performed using citrate buffer (pH 6.0)/EDTA buffer (pH 9.0) under heat-induced conditions. Endogenous peroxidase activity was blocked with 3% hydrogen peroxide. Sections were then incubated overnight at 4 °C with primary antibodies: anti-MT-CO1 (Clone EPR19642; Abcam, Cambridge, United Kingdom; dilution rate, 1:250). Visualization was achieved using a DAB substrate kit, followed by counterstaining with Mayer’s hematoxylin. Paratumoral renal tubules served as the internal positive control for MT-CO1.

### 2.18 Pathological scoring and spatial analysis

The immunohistochemistry (IHC) results were independently evaluated by two senior pathologists blinded to the clinical data.

1. H-score quantification: For MT-CO1, the H-score was calculated using the formula H-score=∑(PI×I), where PI represents the percentage of positive tumor cells (0%–100%) and I represents the staining intensity (0, negative; 1, weak; 2, moderate; and 3, strong).
2. Spatial pattern categorization: Based on the spatial distribution of MT-CO1, samples were categorized into the following: (a) uniform positive, where >80% of the tumor area showed continuous strong staining; b) polarized/mixed, characterized by distinct MT-high and MT-low nests within the same section; and c) negative/fragmented, where MT-CO1 was absent or restricted to small sequestered clusters.
3. Immune infiltration assessment: To evaluate the interaction between metabolic states and the immune microenvironment, we identified large immune aggregates (mononuclear cell clusters with a diameter >200 μm). The frequency of these aggregates was compared between MT-CO1-positive and MT-CO1-negative tumor nests within the same tissue section.

### 2.19 Statistical analysis

All analyses were performed using Python 3.10 and R 4.3.1. Specific statistical methods are detailed in the above corresponding sections. The proportions of tumor subpopulations across different time points were compared using the chi-square test. For all other analyses not explicitly mentioned, the Wilcoxon test or Spearman’s rank correlation was applied.

## 3 Result

### 3.1 Identifying NMA from MT heterogeneity

**Figure 1:**
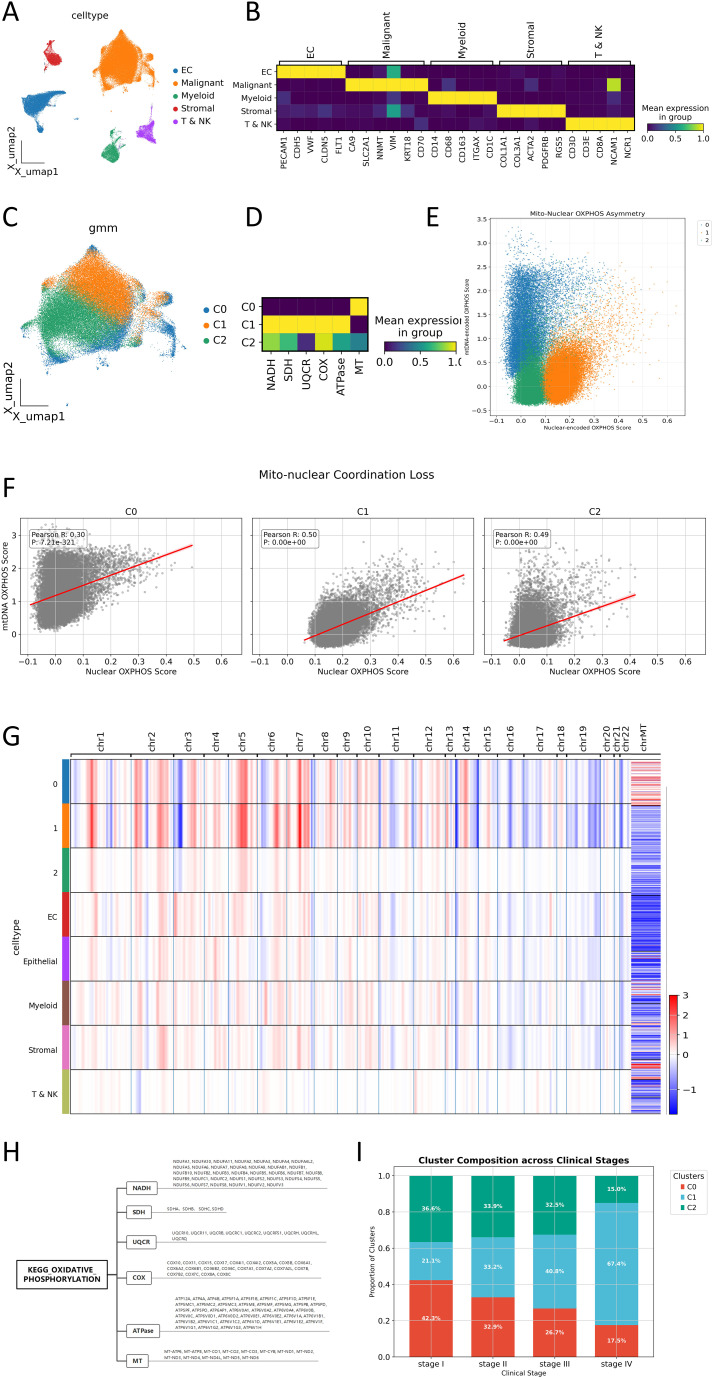
MT gene expression exhibits NMA in ccRCC. **(A)** UMAP distribution of all annotated cell types in ccRCC samples. **(B)** Dot plot showing marker genes for the five major cell populations. **(C)** GMM-based classification of malignant cells into three subpopulations (C0, C1, and C2) using the OXPHOS gene set. **(D)** Heatmap of MT functional gene expression across subpopulations, highlighting the asymmetry in C0. **(E)** Scatter plot of nuclear-encoded versus mtDNA-encoded scores showing polarization of C0 and C1. **(F)** Coordination analysis showing a significant loss of synchrony between nuclear and MT expression in the C0 subpopulation. **(G)** Heatmap showing inferred nuclear and MT CNVs; C0 exhibits a specific increase in mtDNA copy numbers. **(H)** Functional categorization of the OXPHOS gene set into six groups. **(I)** Stacked bar plot showing the proportional changes of clusters across clinical stages; the C0 proportion decreases with tumor progression. MT, mitochondria; NMA, nucleo-mitochondrial expression asymmetry; ccRCC, clear cell renal cell carcinoma; GMM, Gaussian Mixture Model; CNVs, copy number variations.

To investigate MT heterogeneity in ccRCC, we analyzed scRNA-seq data from the TJ-RCC cohort. We identified five major cell populations: malignant, myeloid, NK&T, endothelial, and stromal cells (**Figures 1A, B**). Detailed sub-clustering and marker identification for the immune and stromal populations are provided in the **Supplementary Material** (**Supplementary Figure 1**).

Based on the critical role of MT heterogeneity in ccRCC, we performed GMM on all malignant cells using the OXPHOS gene set. This analysis identified three distinct subpopulations: C0, C1, and C2 (**Figure 1C**). We divided the OXPHOS pathway into six functional modules, including the NADH dehydrogenase, succinate dehydrogenase, ubiquinol-cytochrome *c* reductase, cytochrome *c* oxidase, ATPase, and MT gene families (**Figure 1H**). The first five families encode the major structural subunits of the oxidative phosphorylation complexes I, II, III, IV, and ATP synthase. The MT genes, entirely encoded by the MT genome, participate in the structure of all complexes except complex II (38). Heatmap analysis showed that C0 highly expressed genes encoded by the MT genome, while nuclear-encoded respiratory chain subunits remained low or stable. In contrast, C1 showed higher expression of nuclear-encoded genes (**Figure 1D**). Evidently, a pronounced NMA exists among these subpopulations.

To quantify this NMA, we calculated mtDNA-encoded and nuclear-encoded scores for each cell. Scatter plots revealed a distinct polarization between C0 and C1, with C0 shifting significantly toward the mtDNA-high axis (**Figure 1E**). Coordination analysis further showed that nuclear and MT gene expression remained synchronized in C2 (R = 0.49) and C1 (R = 0.50). However, this coordination significantly decreased in C0 (R = 0.30), suggesting a dysregulated or compensatory hyperactive state of MT function (**Figure 1F**).

We then inferred genomic alterations using inferCNV. These alterations were consistent with those of previous studies, with losses predominantly observed in chr3p, chr14, chr19, and chr22 and gains in chr1, chr2, chr5, and chr7 (7). The results also confirmed that C0 specifically harbored a unique increase in mtDNA CNVs, consistent with its high transcriptional activity (**Figure 1G**). Finally, we examined the proportion of these clusters across clinical stages. Statistical analysis showed that the C0 subpopulation was most enriched in the early stages (Stage I) and declined significantly as the disease progressed to Stage IV (**Figure 1I**).

In summary, these findings identify a specific early-stage subpopulation in ccRCC characterized by NMA and mtDNA amplification. This subpopulation likely plays a key role in tumor initiation and early expansion.

### 3.2 NMA characterizes a high-biosynthetic proliferative engine

**Figure 2:**
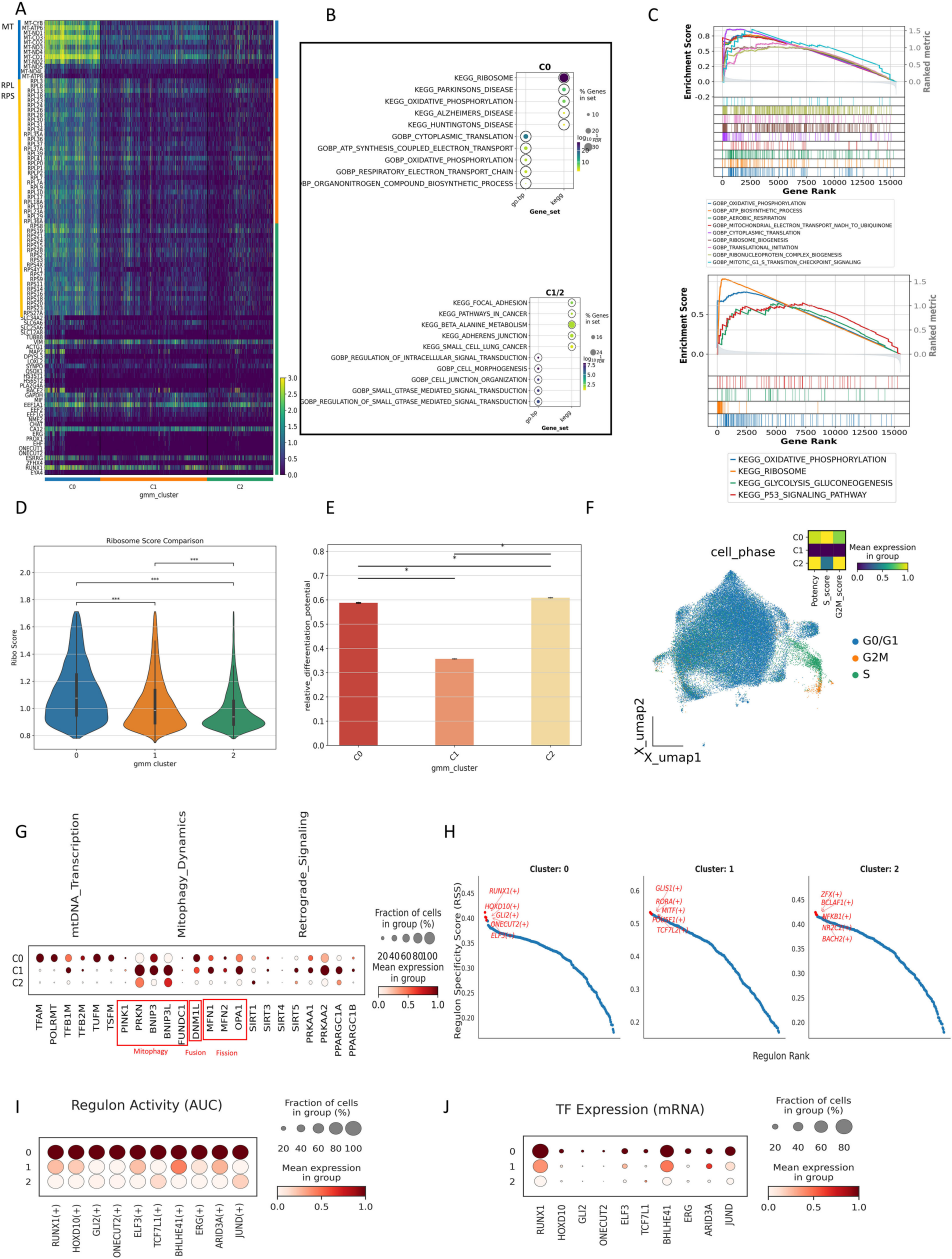
The profound NMA subpopulation functions as a high-biosynthetic proliferative engine. **(A)** Heatmap of differentially expressed genes showing co-activation of MT and ribosomal (RPL/RPS) genes in C0. **(B)** Enrichment analysis (GO BP and Kyoto Encyclopedia of Genes and Genomes (KEGG)) highlighting metabolic and biosynthetic pathways in C0 versus signaling pathways in C1/C2. **(C)** GSEA plots illustrating systemic upregulation of OXPHOS and translation-related gene sets in C0. **(D)** Violin plot comparing ribosome scores across clusters, with C0 exhibiting the highest activity. **(E)** CytoTRACE analysis showing peak differentiation potential and stem-like properties of the C0 subpopulation. **(F)** Cell cycle analysis indicating concentrated G2M- and S-phase activity within the C0 cluster. **(G)** Molecular landscape of mitochondrial regulation across malignant subpopulations. Dot plot showing the expression of genes involved in mtDNA transcription (e.g., *TFAM* and *POLRMT*), mitophagy (e.g., *PRKN* and *BNIP3*), mitochondrial dynamics (fission, *DNM1L*; fusion, *MFN1*, *MFN2*, and *OPA1*), and retrograde signaling (e.g., *SIRT*s, *PRKAA*s, and *PPARGC1A/B*) across malignant clusters C0, C1, and C2. **(H)** RSS identifying master transcription factors (e.g., RUNX1 and GLI2) for each cluster. **(I, J)** Dot plots demonstrating high consistency between TF regulatory activity and mRNA expression levels for C0-specific regulators. NMA, nucleo-mitochondrial expression asymmetry; MT, mitochondria; RPL/RPS, Ribosomal Protein Large/Ribosomal Protein Small; GO, Gene Ontology; BP, Biological Process; GSEA, gene set enrichment analysis; RSS, Regulon Specificity Score; TF, transcription factor. *p < 0.05, ***p < 0.001.

After establishing the NMA of the C0 subpopulation, we further characterized its biological identity. DEGs revealed that C0 co-activated a large set of ribosomal protein genes (RPL/RPS family) alongside its high MT gene expression (**Figure 2A**). Pathway enrichment analysis confirmed that C0 was specifically enriched in “ribosome”, “oxidative phosphorylation”, and “cytoplasmic translation” pathways. In contrast, C1 and C2 clusters favored “focal adhesion” and cancer-related signaling pathways, suggesting a functional shift from metabolism to environmental interaction (**Figure 2B**). GSEA further substantiated the systemic upregulation of ATP biosynthesis and translational initiation machinery in C0, defining it as the primary metabolic hub within the tumor (**Figure 2C**). Quantitative scoring confirmed that C0 possessed the highest ribosomal activity compared to other malignant cells (**Figure 2D**).

We then assessed the developmental status and mitotic activity of these clusters. CytoTRACE analysis identified C0 as having the peak differentiation potential, placing it at the developmental apex of the malignant cell lineage (**Figure 2E**). This high plasticity was coupled with rapid cell division, as evidenced by the high concentration of G2M- and S-phase cells within the C0 cluster (**Figure 2F**). Collectively, these findings define C0 as a “proliferative engine” driving early clonal expansion.

Mechanistic analysis of mitochondrial regulation revealed that the C0 “proliferative engine” is driven by a unique autonomous program (**Figure 2G**). C0 cells specifically upregulated core mtDNA transcription and translation machinery (TFAM, POLRMT, TFB1M/2M, TUFM, and TSFM) while paradoxically suppressing the canonical nuclear-encoded SIRT–AMPK–PGC-1α regulatory axis (SIRT1/3/4/5, PRKAA1/2, and PPARGC1A/B). This “bottom-up” decoupling was further supported by a distinct pro-fission (high DNM1L), low-fusion (MFN1/2 and OPA1), and low-mitophagy (PRKN and BNIP3/3L) transcriptional signature in C0. These findings suggest that C0 cells structurally preserve a fragmented, hyperactive mitochondrial pool to fuel intensive biosynthetic output and rapid mitotic activity.

Finally, we explored the transcriptional landscape driving this engine-like state using pySCENIC. The regulon specificity score (RSS) identified master regulators such as RUNX1, GLI2, and ELF3 as the primary drivers of C0 (**Figure 2H**). The regulatory activities [area under the curve (AUC)] of these transcription factors were highly consistent with their mRNA levels (**Figures 2I, J**). Given the roles of RUNX1 and GLI2 in maintaining stem-like states and promoting cell cycle progression, these factors likely coordinate the high metabolic and proliferative output observed in C0 (39, 40).

In summary, the C0 subpopulation represents a unique functional entity in ccRCC that couples MT energy production with intensive protein biosynthesis to fuel tumor initiation and rapid growth.

### 3.3 TCA cycle metabolite accumulation supports the proliferative phenotype

**Figure 3:**
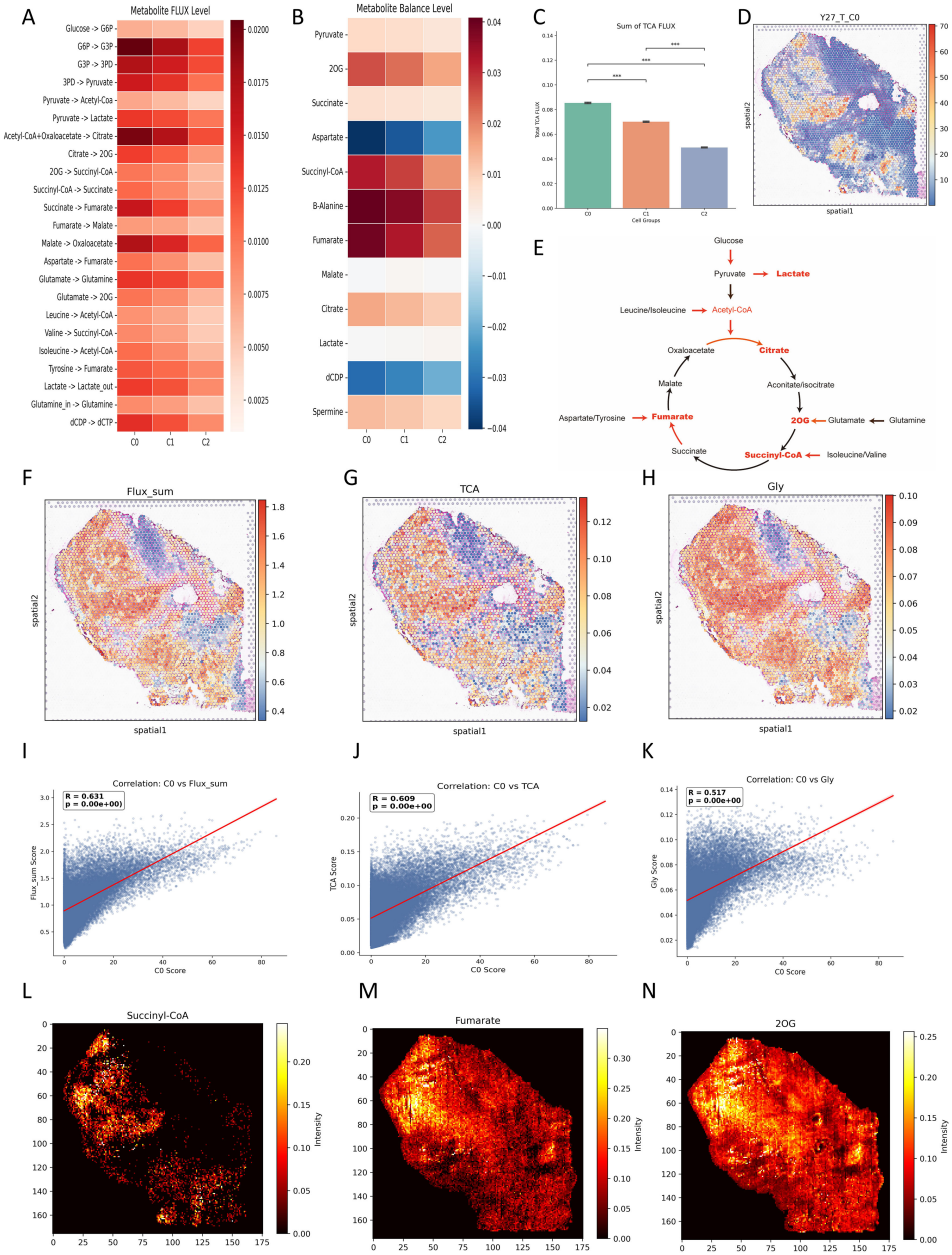
TCA cycle metabolite accumulation supports biosynthesis and proliferation. **(A)** Heatmap of inferred metabolic fluxes across malignant subpopulations. **(B)** Heatmap of metabolite balances, where red indicates accumulation and blue indicates depletion. **(C)** Bar plot comparing total TCA cycle flux showing C0 as the most metabolically active cluster. **(D)** Spatial distribution of the C0 subpopulation in sample Y27_T, serving as a localization reference. **(E)** Schematic summarizing the metabolic rewiring (glycolysis, TCA, and amino acid catabolism) in C0 cells. **(F–H)** Spatial maps showing the predicted distribution of total metabolic flux, TCA cycle activity, and glycolysis. **(I–K)** Correlation scatter plots demonstrating the high spatial consistency between the C0 score and metabolic activities. **(L–N)** MSI of spatial metabolomics showing the actual physical distribution of succinyl-CoA, fumarate, and 2OG, which align with the C0-rich areas. TCA, tricarboxylic acid; MSI, mass spectrometry imaging. ***p < 0.001.

To determine whether the NMA signatures of C0 translate into functional metabolic advantages, we employed scFEA to infer metabolic flux and metabolite balances. At the single-cell level, the C0 subpopulation exhibited significantly higher flux across most tricarboxylic acid (TCA) cycle steps compared to the C1 and C2 subpopulations (**Figure 3A**). Analysis of metabolite balances revealed a marked accumulation of key intermediates, including 2-oxoglutarate (2OG), succinyl-CoA, and fumarate within C0 (**Figure 3B**). Quantitative comparison confirmed that C0 possessed the highest total TCA cycle flux, reinforcing its role as the tumor’s primary metabolic hub (**Figure 3C**). This hypermetabolic state is supported by a coordinated rewiring of energy pathways. Specifically, while ccRCC cells maintain high glycolytic flux to lactate, the C0 subpopulation uniquely sustains its TCA cycle through the catabolism of branched-chain amino acids (leucine/isoleucine) (BCAAs) and glutamine (**Figure 3E**). We then mapped these metabolic programs onto the tissue architecture. In representative sample Y27_T, the C0 subpopulation was precisely localized to the dense tumor core (**Figure 3D**). Spatial scFEA modeling revealed that the total metabolic activity (Flux sum), TCA cycle intensity, and glycolytic activity were all highly concentrated within these C0-dominant regions (**Figures 3F–H**). Quantitative correlation analysis at the spot level confirmed that the C0 score was strongly and positively correlated with total metabolic flux (R = 0.631), TCA intensity (R = 0.609), and glycolysis (R = 0.517) (**Figures 3I–K**). These correlations suggest that C0 cells are the primary spatial carriers of high-energy metabolism.

To provide direct biochemical evidence, we utilized MSI-based spatial metabolomics to measure the actual distribution of metabolites. Strikingly, the physical accumulation of TCA intermediates—succinyl-CoA, fumarate, and 2OG—closely mirrored the predicted metabolic hotspots and the spatial distribution of the C0 subpopulation (**Figures 3L–N**). The precise co-localization of these metabolites with the MT-high subpopulation confirms that C0 acts as a “proliferative engine”. In this context, NMA is characterized by the utilization of high MT output to generate the energy and biosynthetic precursors required for rapid tumor expansion.

### 3.4 Proliferative phenotype evolves toward a hypoxic/immune-evasive state coupled with NMA attenuation

**Figure 4:**
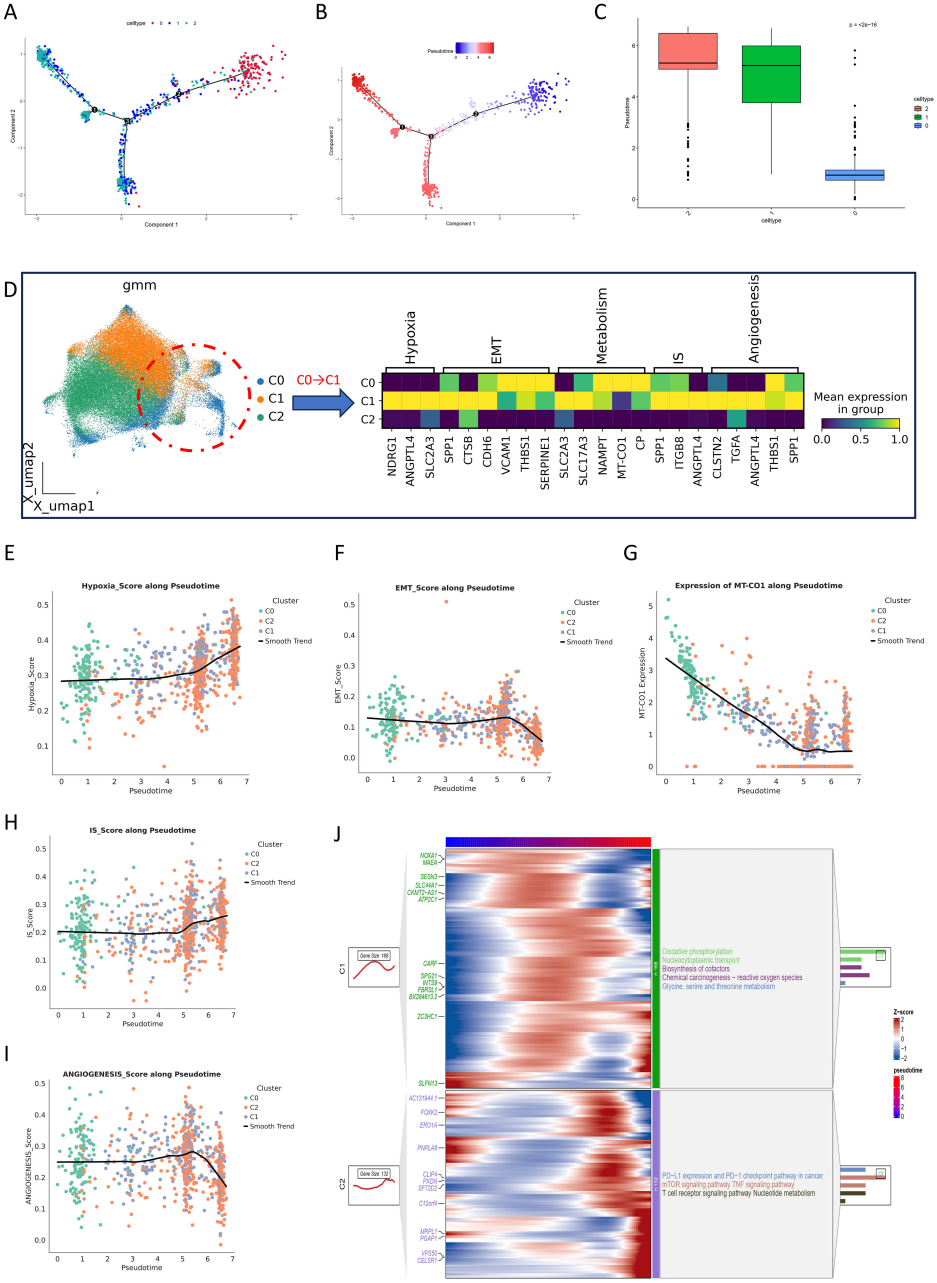
Proliferative phenotype evolves toward a hypoxic/immune-evasive state coupled with NMA exhaustion. **(A, B)** Pseudotime trajectory of malignant cells showing C0 at the root and C1/C2 at the terminal branches. **(C)** Box plot comparing pseudotime values across the three malignant clusters (*p* < 2e−16). **(D)** Heatmap of key gene expression changes during the C0-to-C1 transition, categorized by functional modules. **(E–I)** Dynamic scores for hypoxia, EMT, mitochondrial metabolism (*MT-CO1*), immune suppression (IS), and angiogenesis along the pseudotime axis. **(J)** Clustered heatmap of genes significantly associated with pseudotime, with early stages enriched for metabolism and late stages for immune modulation pathways. TCA, tricarboxylic acid; MSI, mass spectrometry imaging; NMA, nucleo-mitochondrial expression asymmetry; EMT, epithelial–mesenchymal transition.

To understand the dynamic relationship between the identified subpopulations, we performed pseudotime trajectory analysis using Monocle2. The malignant cell population exhibited a clear bifurcating developmental path, with the C0 subpopulation consistently positioned at the root of the trajectory (**Figures 4A, B**). Quantitative comparison of pseudotime values confirmed that C0 occupied the earliest developmental state, transitioning toward the more differentiated C1 and C2 states (**Figure 4C**).

We further investigated the molecular programs driving the transition from C0 to C1. Analysis of highly variable genes along this trajectory revealed a coordinated “phenotypic switch” (**Figure 4D**). Specifically, as cells progressed along the pseudotime axis, there was a significant downregulation of MT metabolism genes (e.g., *MT-CO1* and *NAMPT*) and ribosomal programs. Concurrently, the expression of hypoxia-response genes (*NDRG1* and *ANGPTL4*) and immune suppression (IS) markers (*SPP1* and *ITGB8*) showed a progressive upward trend (**Figures 4E, H**). Interestingly, markers of epithelial–mesenchymal transition (EMT) and angiogenesis exhibited transient fluctuations but generally trended downward in the terminally differentiated C1 state compared to C0. This downregulation suggests that C1 represents a more specialized, metabolically dormant but immune-evasive phenotype (**Figures 4F, I**).

Functional enrichment of genes across the pseudotime revealed two distinct waves of biological activity (**Figure 4J**). The early stage (dominated by C0) was highly enriched for OXPHOS and nucleocytoplasmic transport, supporting the “proliferative engine” model. In contrast, the late stage (dominated by C1) was characterized by the activation of PD-L1/PD-1 checkpoint pathways and TNF signaling. This transition represents a strategic shift from rapid biomass production to immune landscape remodeling (41, 42). These findings suggest that ccRCC cells undergo a systematic “metabolic shedding” under the influence of NMA. In this process, they sacrifice MT efficiency to prioritize enhanced survival and immune escape under environmental stress.

### 3.5 NMA reveals subpopulation exclusion and evolving neighborhood dynamics

**Figure 5:**
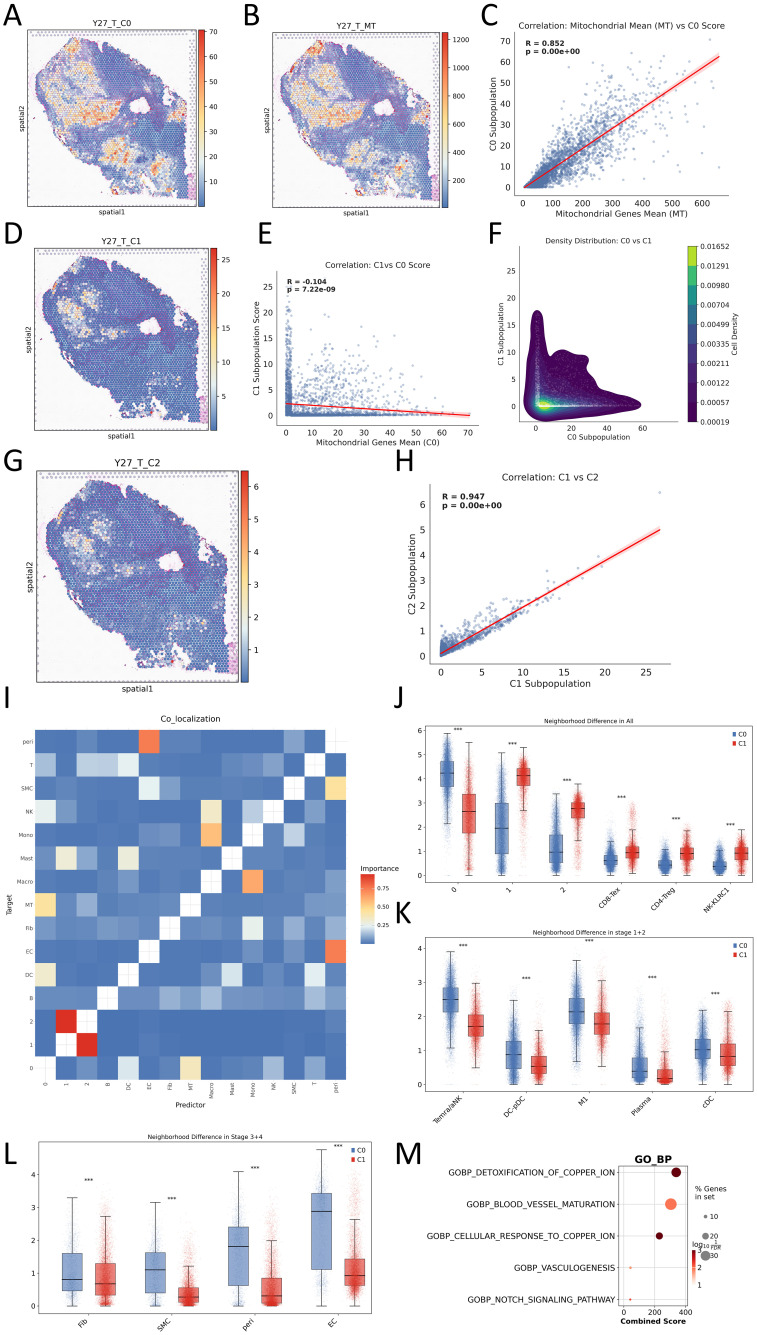
NMA reveals subpopulation exclusion and evolving neighborhood dynamics. **(A–C)** Spatial mapping and correlation scatter plot showing precise co-localization between C0 and MT gene expression. **(D–F)** Spatial maps and density distribution revealing the mutual exclusivity and L-shaped trade-off between C0 and C1 clusters. **(G, H)** Spatial overlap and strong linear correlation between C1 and C2 subpopulations. **(I)** Heatmap of co-localization importance scores among all cell types in the tumor microenvironment. **(J)** Neighborhood analysis across all stages showing higher proximity of C1 to immunosuppressive immune cells. **(K, L)** Stage-specific neighborhood comparison showing C0 transitioning from immune-rich environments in early stages to stroma-rich environments in advanced stages. **(M)** Dot plot of GO Biological Process enrichment for the genes localized within the C0 spatial neighborhood. NMA, nucleo-mitochondrial expression asymmetry; MT, mitochondria; GO, Gene Ontology. ***p < 0.001.

To characterize the influence of NMA in the spatial architecture, we mapped the subpopulations with NMA onto tissue space. In representative sample Y27_T, the C0 subpopulation demonstrated precise spatial alignment with high MT gene expression (**Figures 5A, B**; **Supplementary Figure 2**). Quantitative analysis at the spot level confirmed a strong linear correlation between C0 abundance and MT gene scores (R = 0.852), validating C0 as the primary carrier of NMA (**Figure 5C**). In contrast, the C1 subpopulation occupied distinct geographic regions, exhibiting significant spatial exclusivity with C0 (**Figures 5D, E**; **Supplementary Figure 3**). Density distribution analysis revealed a characteristic L-shaped pattern, suggesting that C0 and C1 rarely co-exist within the same spatial spot (**Figure 5F**). Conversely, C1 and C2 showed high spatial overlap and strong linear correlation (R = 0.947), forming a combined niche (**Figures 5G, H**).

Beyond malignant cells, we explored the global co-localization network, such as the co-localization of macrophages with monocytes and stromal cells with endothelial cells (ECs) (**Figure 5I**). We then employed neighborhood analysis to quantify the cellular “social circles” of C0 and C1. While both subpopulations exhibited strong self-aggregation, C1 was more frequently neighbored by immunosuppressive cells, including CD8+ exhausted T cells (Tex), CD4+ regulatory T cells (Treg), and NK-KLRC1 cells (**Figure 5J**).

Crucially, the neighborhood of C0 underwent a dramatic shift during disease progression. In the early stages (Stages I–II), C0 was predominantly associated with active immune infiltrates, including Temra/aNK, plasmacytoid dendritic cells (Pdc), and M1 macrophages, suggesting an “immune-active” core (**Figure 5L**). However, in the advanced stages (Stages III–IV), the C0 neighborhood became increasingly dominated by fibroblasts, smooth muscle cells (SMCs), and pericytes (**Figure 5K**). Pathway enrichment of C0-adjacent regions revealed significant activity in copper ion detoxification and cellular response. This finding aligns with the high MT demand of C0 cells, while also highlighting the enrichment of Notch signaling and vasculogenesis pathways (**Figure 5M**). As the disease advances, C0 cells transition from an immune-engaged state to a stroma-sequestered state. This progression suggests that NMA exerts a significant influence on the spatial niche, driving the reconstruction of the cellular neighborhood.

### 3.6 Spatial evolution of NMA: niche reconfiguration from “core” to “island”

**Figure 6:**
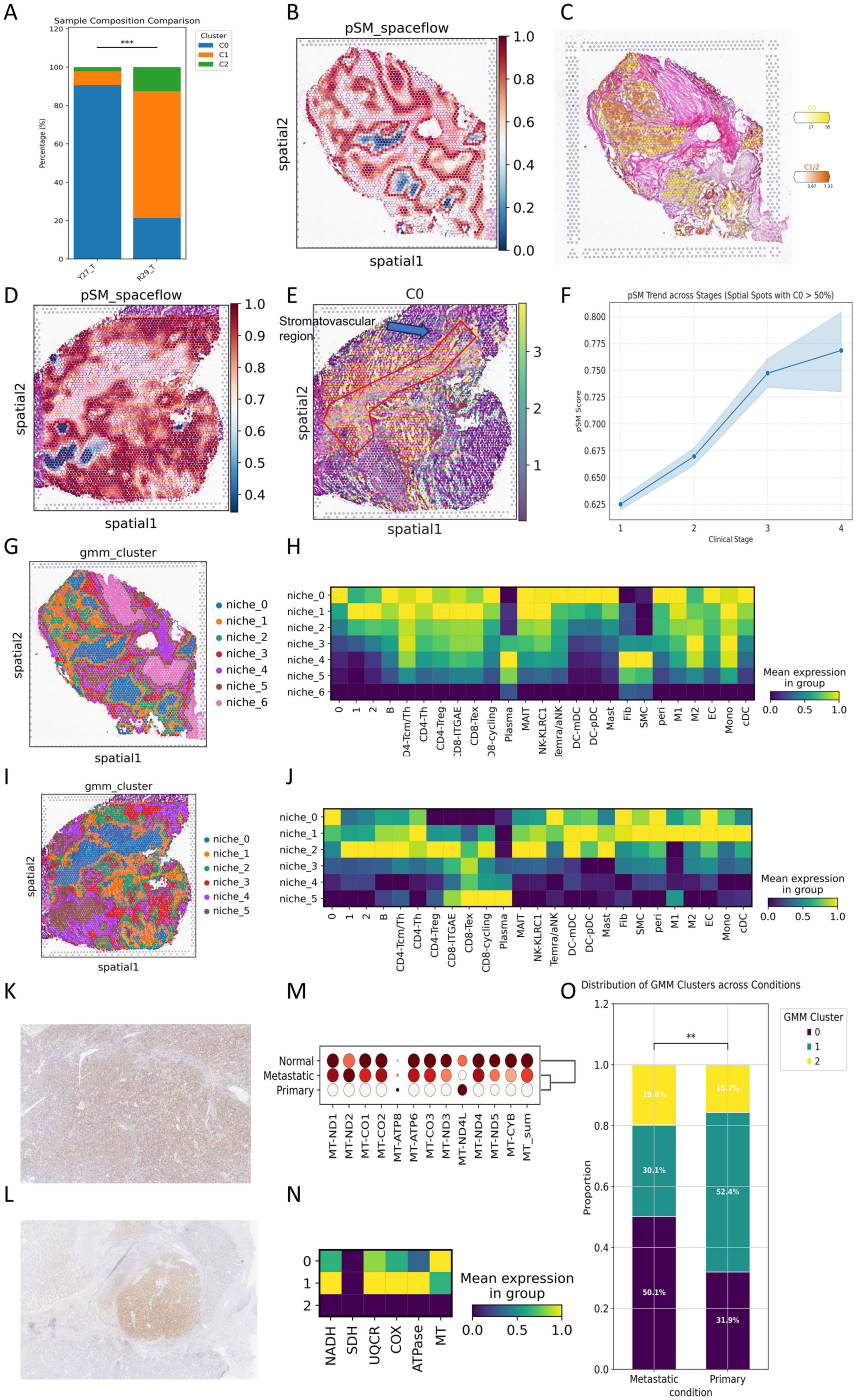
Spatial evolution of NMA: niche reconfiguration from “core” to “island”. **(A)** Subpopulation proportions in early (Y27_T) versus late-stage (R29_T) samples. **(B, C)** pSM and C0 mapping in Y27_T showing co-localization of C0 with complex infiltration zones (low pSM signal). **(D, E)** pSM and C0 mapping in R29_T showing sequestration of C0 in purified zones (high pSM signal) and infiltration into stroma. **(F)** Line plot showing the progressive increase of average pSM scores in C0-dominant spots across clinical stages. **(G, H)** Niche clustering and cell distribution matrix for Y27_T, highlighting immune-active cores. **(I, J)** Niche clustering and cell distribution matrix for R29_T highlighting stroma-sequestered islands. **(K, L)** Representative MT-CO1 IHC images of early-stage (uniform core) and late-stage (isolated island) ccRCC. **(M)** Dot plot of mitochondrial gene expression across normal, primary, and metastatic samples. **(N)** Heatmap of OXPHOS family expression in the validation cohort showing consistent nucleo-mitochondrial asymmetry. **(O)** Proportion of malignant clusters in primary versus metastatic lesions showing enrichment of C0 in metastatic nests. NMA, nucleo-mitochondrial expression asymmetry; pSM, pseudo-spatiotemporal map; IHC, immunohistochemistry; ccRCC, clear cell renal cell carcinoma. **p < 0.01, ***p < 0.001.

Our previous findings suggested a systematic remodeling of the spatial neighborhood under the influence of NMA. To gain a more systematic understanding of how NMA influences the spatial ecology, we selected two representative cases for in-depth analysis. These include Y27 and R29, which serve as classic benchmarks for the early and late stages of ccRCC, respectively. Comparison of cluster proportions between early-stage (Y27_T) and late-stage (R29_T) samples revealed that Y27_T was predominantly composed of C0 cells (>80%), while R29_T was dominated by the invasive C1 cluster (**Figure 6A**). To explore the underlying spatial logic, we utilized SpaceFlow to generate pSM. In early-stage Y27_T, the C0 subpopulation localized to regions with low pSM signals, reflecting a high degree of gene expression complexity due to intense cell–cell interactions and immune infiltration (**Figures 6B, C**). In contrast, in late-stage R29_T, C0 was found in high-signal pSM regions, indicating a more homogeneous and purified cancer cell state. Furthermore, low-signal pSM areas shifted outward beyond the tumor region, and C0 exhibited significant infiltration into stroma-rich vascularized zones (**Figures 6D, E**). Quantitative analysis confirmed that the average pSM score for C0-dominant spots increased progressively from Stage I to Stage IV. This trend indicates that NMA systematically influences the spatial landscape, leading to the “purification” or isolation of the C0 niche during tumor evolution (**Figure 6F**).

We further defined functional niches based on the pSM and NMA. In the early-stage sample, Niche 0 (dominated by C0) was characterized by the co-localization of C0 with substantial immune cells (T cells and B cells) and endothelial cells (**Figures 6G, H**). In the late-stage sample, however, the C0 niche (Niche 0) underwent significant sequestration. Specifically, it was composed mainly of cancer cells and stromal elements, with immune effectors restricted to the surrounding peripheral layers (**Figures 6I, J**). This physical transition was histologically confirmed by MT-CO1 IHC staining, which revealed uniform, continuous high-density cores of MT-CO1+ cells in early-stage tumors. In contrast, advanced samples exhibited fragmented and sequestered “islands” encased in a thick, fibrotic stroma (**Figures 6K, L**).

**Figure 7:**
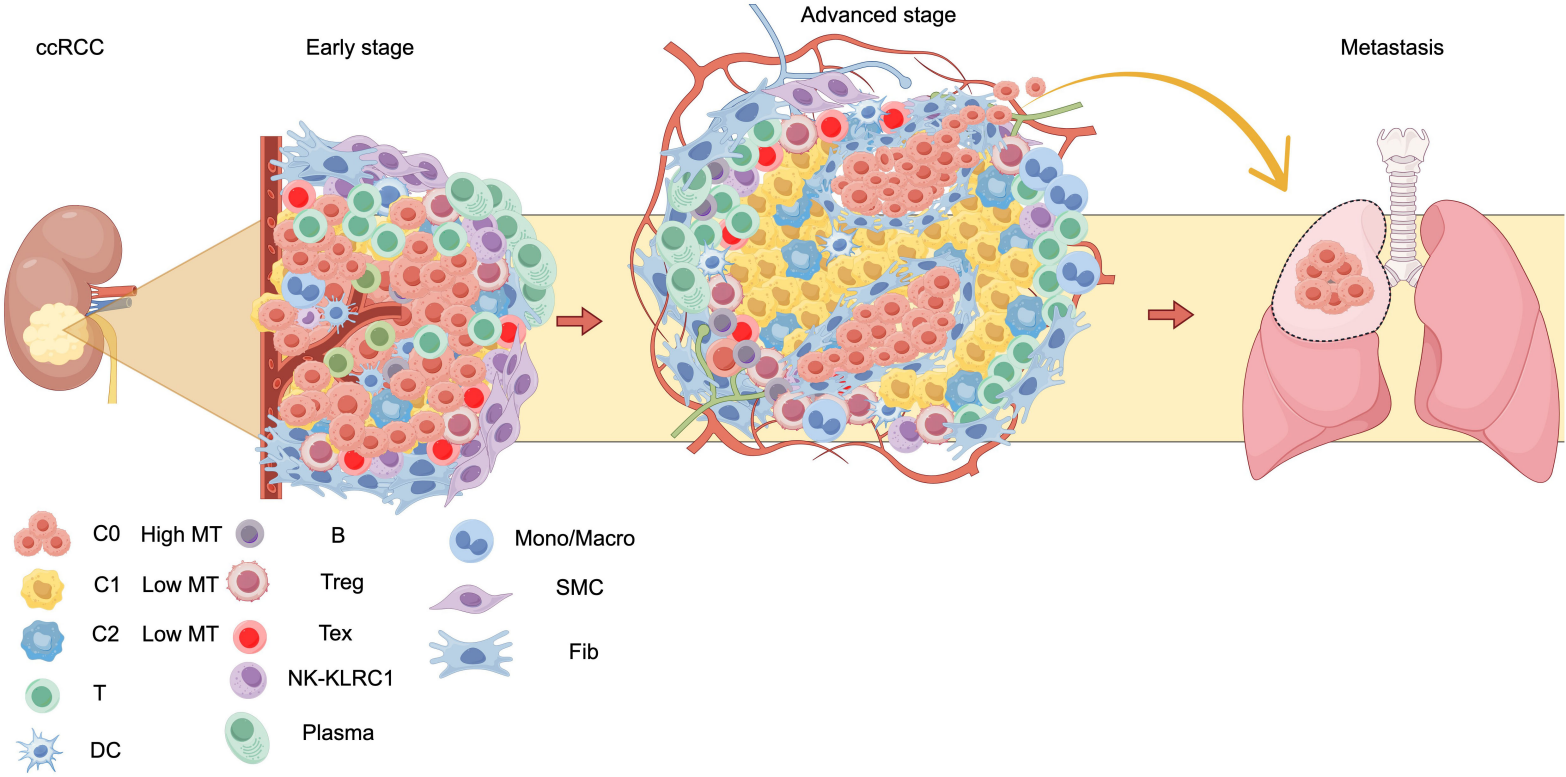
Working model of NMA in ccRCC progression. A spatial framework illustrating the evolution of NMA niches. (Early stage) NMA-high C0 malignant cells function as a “proliferative engine” in an immune-active central core, characterized by high cell–cell interactions with active immune effectors (T cells, B cells, and DCs). (Advanced stage) As the tumor progresses, C1/C2 clusters expand, and C0 cells are sequestered into fragmented “metabolic islands” shielded by a thick fibrotic stroma (SMC, Fib) and vascularized niches (pericytes and endothelial cells). (Metastasis) Upon systemic dissemination, the NMA-high “proliferative engine” (C0) resurges to seed and fuel the expansion of metastatic colonies in distant organs such as the lung. NMA, nucleo-mitochondrial expression asymmetry; ccRCC, clear cell renal cell carcinoma; DC, dendritic cell; SMC, smooth muscle cell; Fib, fibroblast.

Finally, we extended our study to a paired metastatic cohort (normal, primary, and metastatic samples). Interestingly, MT genes, which diminish in advanced primary tumors, showed a dramatic resurgence in metastatic lesions (**Figure 6M**). The metastatic malignant cells displayed the same NMA observed in our discovery cohort (**Figure 6N**). Importantly, the proportion of the C0 subpopulation was significantly higher in metastatic nests compared to primary tumors (**Figure 6O**). This striking enrichment suggests that the proliferative engine is strategically reactivated to seed and fuel the growth of new colonies during systemic dissemination. These findings across different stages establish a systematic spatial-metabolic framework for the evolution of NMA (**Figure 7**).

### 3.7 MT-CO1 as a histological proxy for NMA: large-scale clinical validation, prognostic prediction, and drug prediction

**Figure 8:**
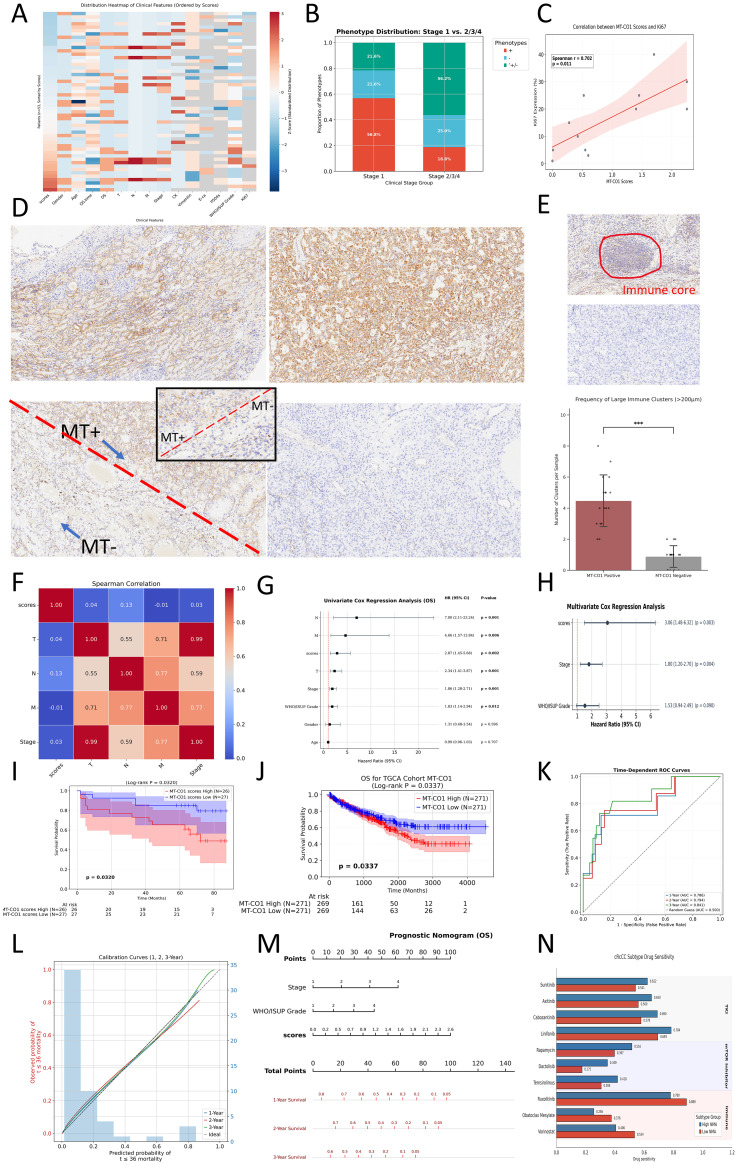
Large-scale clinical validation and prognostic prediction based on MT-CO1. **(A)** Heatmap summarizing clinical characteristics and H-scores of the validation cohort (n = 53). **(B)** Stacked bar plot showing the distribution of MT-CO1 phenotypes (positive, polarized, and negative) across clinical stages. **(C)** Correlation between MT-CO1 H-score and Ki67 index, highlighting the proliferative trait of C0 cells. **(D)** Representative IHC images showing the evolutionary sequence: paratumoral tubule (strong positive) → early cancer (uniform positive) → transition (intra-nest polarization) → advanced cancer (negative). **(E)** Comparison of large immune cluster (>200 μm) frequency between MT-positive and MT-negative nests. **(F–H)** Statistical validation of H-score: Spearman’s correlation with clinical factors **(F)**, univariate Cox **(G)**, and multivariate Cox **(H)** regression analyses. **(I, J)** Kaplan–Meier survival curves based on MT-CO1 levels in the validation cohort **(I)** and TCGA-KIRC cohort **(J)**. **(K, L)** Performance evaluation of the prognostic model: time-dependent ROC curves **(K)** and calibration plots **(L)** for 1-, 2-, and 3-year survival. **(M)** Nomogram for predicting overall survival based on integrated clinical and mitochondrial pathological features. **(N)** Predicted drug sensitivity profiles across NMA subpopulations. Note the preferential sensitivity of the high NMA subpopulation to TKI and mTOR-targeted therapies. IHC, immunohistochemistry; ROC, receiver operating characteristic; NMA, nucleo-mitochondrial expression asymmetry; TKI, tyrosine kinase inhibitor.

Our multi-dimensional -omics analysis revealed a global NMA activation of the MT genome. However, clinical application requires a single, reliable histological marker. Given that mtDNA is transcribed as a polycistronic unit, we selected MT-CO1 as a representative proxy for this metabolic state. We then evaluated its clinical characteristics in an independent cohort of 53 ccRCC patients (**Figure 8A**). Systematic pathological assessment revealed that MT-CO1 staining patterns shifted significantly during disease progression. In early-stage samples, MT-CO1 displayed a predominantly uniform positive (+) pattern, mirroring the high MT density of paratumoral renal tubules (**Figures 8B, D**). However, advanced-stage tumors were characterized by a significant decrease in uniform positivity and an increase in complex “polarized” patterns (+/−), where MT-high and MT-low cells co-existed within the same nest. This heterogeneity ultimately culminated in a complete loss of MT-CO1 expression in terminal stages (**Figures 8B, D**). This evolutionary sequence suggests that ccRCC cells initially retain the metabolic “memory” of the parent renal epithelium but undergo systematic “MTshedding” during progression.

Functional validation in tissue sections confirmed that MT-CO1 expression was positively correlated with the proliferation marker Ki67 (r = 0.702, **Figure 8C**), supporting the “proliferative engine” hypothesis. Intriguingly, large immune aggregates (>200 μm) were significantly more frequent in MT-positive regions compared to MT-negative nests in mixed-expression samples (**Figure 8E**). This observation reinforces our earlier conclusion regarding C0 immune infiltration, suggesting that these cells serve as metabolic beacons that drive localized immune engagement.

Statistical analysis identified the MT-CO1 H-score as a powerful prognostic marker. Univariate and multivariate Cox regression analyses confirmed that the H-score is an independent prognostic factor for overall survival, alongside TNM stage and WHO/ISUP grade (**Figures 8F–H**). The Kaplan–Meier analysis demonstrated that high MT-CO1 expression was significantly associated with poorer survival in our validation cohort, a trend consistently observed in the large-scale TCGA-KIRC dataset (**Figures 8I, J**). To enhance clinical translatability, we developed a prognostic nomogram integrating H-score, stage, and grade (**Figure 8M**). The model exhibited robust predictive accuracy for 1-, 2-, and 3-year survival, as evidenced by high AUC values in time-dependent receiver operating characteristic (ROC) curves and excellent alignment in calibration plots (**Figures 8K, L**). These results establish NMA as a clinically actionable marker for risk stratification in ccRCC.

In addition, drug sensitivity prediction revealed distinct therapeutic vulnerabilities associated with NMA status (**Figure 8N**). The high NMA subpopulation exhibited significantly superior sensitivity to tyrosine kinase inhibitors (TKIs) (e.g., sunitinib, axitinib, and cabozantinib) and mTOR inhibitors (e.g., rapamycin and temsirolimus) compared to the low NMA group. Conversely, high NMA cells showed lower predicted responsiveness to immune-related or other agents, such as ruxolitinib and vorinostat. These findings identify NMA as a potential predictive biomarker for prioritizing TKI and mTOR-targeted regimens in aggressive ccRCC niches, while suggesting relative resistance to alternative therapies.

## 4 Discussion

### 4.1 The biological essence of NMA in ccRCC

Traditionally, ccRCC has been characterized as a predominantly glycolytic tumor where the VHL–HIF axis systematically suppresses MT function (4, 5). However, our multi-omics analysis reveals a complex MT heterogeneity landscape that challenges this monolithic view. We conclude that a specific malignant subpopulation, C0, exhibits profound NMA. Specifically, this state is characterized by a dysregulated burst of mtDNA-encoded transcripts that remains independent of nuclear-encoded respiratory subunits. This conclusion is supported by the significant decrease in nucleo-mitochondrial coordination in C0 (R = 0.30) and a subpopulation-specific increase in mtDNA CNVs.

Crucially, our data provide preliminary insights into the molecular drivers of this asymmetry. We observed that NMA is likely propelled by the targeted upregulation of the core mitochondrial transcription and translation machinery, including TFAM, POLRMT, TFB1M/2M, and the elongation factor TUFM/TSFM. Paradoxically, this mitochondrial-level activation occurs while canonical nuclear-encoded regulators [specifically the SIRT1/3/4/5 family and AMPK (PRKAA1/2)] and the biogenesis master regulators PGC-1α/β (PPARGC1A/B) remain suppressed. This unique molecular signature suggests that NMA is not merely a passive byproduct but an autonomous “bottom-up” program. In this model, the mitochondria appear to bypass the traditional nuclear-encoded “governor” (the SIRT–AMPK–PGC1α axis) that typically limits mitochondrial output under stress. We speculate that this decoupling may be a strategic response to localized redox imbalances or NAD^+^/NADH fluctuations, allowing C0 cells to trigger a “HIF-bypass” mechanism. This state provides a selective bioenergetic advantage during critical periods of tumor expansion (43), enabling these cells to resolve the metabolic paradox of sustaining high biosynthetic output within a pseudohypoxic framework. In addition, our data demonstrate that NMA is not a static feature but follows a dynamic evolutionary trajectory. In primary tumors, this state is predominantly enriched in early-stage (Stage I) lesions and declines significantly as the disease progresses to Stage IV. Crucially, however, we observed a dramatic resurgence of MT gene expression and NMA in metastatic lesions. We conclude that metastatic malignant cells re-adapt NMA. This NMA resurgence is evidenced by a significantly higher abundance of the “proliferative engine” subpopulation in metastatic nests than in advanced primary tumors. Consequently, NMA appears not as a terminal metabolic state but as a highly plastic and tunable program, enabling ccRCC cells to toggle their bioenergetic capacity to navigate stage-specific microenvironmental challenges.

### 4.2 NMA defines a high-biosynthetic proliferative engine

Beyond its unique genomic signature, we find that the C0 subpopulation, characterized by profound NMA, functions as a “proliferative engine” driving early tumor growth. This functional identity is established by the coordinated co-activation of MT transcripts and the cytoplasmic translational machinery (43). Our results demonstrate that C0 cells specifically enrich for “ribosome” and “cytoplasmic translation” pathways, exhibiting a systemic upregulation of RPL/RPS family genes. This high-biosynthetic state is closely coupled with rapid mitotic activity. Specifically, the C0 cluster exhibits a high concentration of G2M/S-phase cells and is classified at the apex of developmental potential via CytoTRACE 2.

Our findings further conclude that this proliferative engine is supported by a significant accumulation of TCA cycle intermediates. scFEA-based flux inference and spatial metabolomics provide direct evidence that key metabolites—specifically 2OG, succinyl-CoA, and fumarate—are physically concentrated within C0-dominant regions. The correlation analysis confirms that C0 abundance is a robust predictor of total metabolic flux, reinforcing its role as the tumor’s primary metabolic hub.

Additionally, this “proliferative engine” appears to be structurally reinforced by a distinct reconfiguration of mitochondrial dynamics. Our data show a trend toward a pro-fission (high fragmentation), low-fusion, and low-autophagy profile in these cells. It is tempting to speculate that such a fragmented mitochondrial network may facilitate the rapid partitioning of mitochondrial units during mitosis, while the observed low-autophagy state could theoretically prevent the degradation of these hyperactive mitochondria, thereby stabilizing the NMA state. These integrated results suggest that NMA provides the bioenergetic foundation required for such intensive biosynthetic output. We contend that bypassing nuclear-encoded metabolic constraints enables C0 cells to generate surplus ATP and carbon skeletons, fueling the rapid biomass production identified in our trajectory analyses (44). In this context, the accumulation of TCA intermediates is not merely a metabolic byproduct, but a hallmark of the high-flux energy state that fuels early clonal expansion.

### 4.3 Spatial NMA evolution: niche reconfiguration from “core” to “island”

A central contribution of this study is the characterization of NMA within the macroscopic tissue architecture. We conclude that the C0 subpopulation is the primary spatial carrier of NMA, demonstrated by the strong linear correlation between C0 abundance and MT genes (R = 0.852). Our results reveal a striking spatial exclusivity between the profound NMA “proliferative engine” (C0) and the differentiated, immune-evasive C1/C2. This geographic separation suggests that NMA defines a distinct spatial niche that is incompatible with hypoxic phenotypes of late-stage disease (45).

Our data further demonstrate that the cellular “neighborhood” of the profound NMA cells undergoes a systematic reconfiguration tracked by pSM. In early-stage (Stage I–II) samples, C0 cells localize to low-signal pSM regions, reflecting a state of high gene expression complexity and active immune engagement. Conversely, the pSM for C0-dominant spots increases significantly in the advanced stages (Stage III–IV), indicating a transition to a more purified and isolated cancer state. We conclude that this transition is marked by the reconstruction of the “immune-active core” into sequestered “metabolic islands”. Our spatial analysis reveals that these late-stage islands infiltrate stroma-rich vascularized zones (46, 47), characterized by pericyte enrichment and active Notch signaling (48). This recruitment of vascularized stroma serves as the structural foundation for systemic dissemination (49, 50).

These findings suggest that the spatial evolution of NMA reflects a process of niche specialization in response to microenvironmental pressure. In the early stages, the profound NMA “engine” state is sustainable within the tumor core near active immune infiltrates. However, as the microenvironment becomes increasingly hostile, an intricate immunometabolic reconfiguration takes place. NMA-driven metabolic rewiring may actively shape this landscape: high metabolic flux within C0-dominant regions could induce localized nutrient competition and immunosuppressive metabolite accumulation, potentially driving TAM polarization and T-cell exhaustion. In this model, the transition to fragmented “metabolic islands” represents a strategic adaptation where NMA cells dock onto stroma-rich vascular interfaces. This niche secures the critical oxygen and nutrient supply required for high bioenergetic output while simultaneously creating an “immunometabolic sanctuary” to evade immune clearance. Such spatial partitioning not only facilitates localized persistence but also serves as the structural foundation for metastatic initiation.

### 4.4 Clinical translation: MT-CO1 as a spatio-metabolic readout of progression and therapeutic response

We sought to translate the multi-dimensional NMA signature into a clinically actionable tool by evaluating MT-CO1—a core catalytic subunit encoded by the MT genome—as a histological proxy for NMA. Validation in our independent cohort (n = 53) and the TCGA-KIRC dataset reveals that MT-CO1 protein expression provides a robust, stage-specific readout of ccRCC evolution. Early-stage tumors are predominantly characterized by uniform MT-CO1 positivity, whereas advanced-stage lesions frequently exhibit complex polarized or mixed patterns. This heterogeneity, marked by the coexistence of MT-high and MT-low nests and a progression toward total expression loss, provides clear evidence for systematic “MT shedding” as cells transition toward a more glycolytic phenotype (51).

Our clinical analysis further validates the functional identity of the profound NMA state. The strong positive correlation between MT-CO1 H-scores and the proliferation marker Ki67 (r = 0.702) reinforces the “proliferative engine” model. Crucially, we observed that large immune aggregates (>200 μm) are significantly more frequent in MT-CO1-positive regions compared to negative nests within the same tumor section. This spatial evidence establishes that the NMA captured by our multi-omics analysis indeed translates into a physical “proliferative engine” that is intimately coupled with the local immune landscape. Statistically, we conclude that the MT-CO1 H-score is a powerful and independent prognostic marker associated with poorer overall survival.

Beyond its prognostic value, NMA serves as a predictive biomarker for therapeutic response, reflecting the broader context of cancer metabolic plasticity (10). Our drug sensitivity analysis reveals that the high NMA subpopulation exhibits significantly increased sensitivity to TKIs such as sunitinib and axitinib, as well as mTOR pathway inhibitors (e.g., rapamycin), which act downstream of the HIF-2α axis. Conversely, high NMA cells may exhibit intrinsic resistance to immune checkpoint inhibitors (ICIs). This potential resistance is of particular clinical importance: the “metabolic islands” characterized by high NMA and dense MT-CO1 expression likely create an “immunometabolic sanctuary”. Within these niches, high metabolic flux and subsequent nutrient competition may render ICBs less effective compared to other malignant clusters.

Finally, it is essential to acknowledge that the biological role of mitochondrial activity is context-dependent. As highlighted by recent studies, mitochondrial signaling can exert dual roles—acting as either a tumor suppressor or promoter—depending on the tumor stage and specific metabolic landscape. By integrating the MT-CO1 H-score with traditional TNM staging into a prognostic nomogram, we provide a refined framework for risk stratification. This approach facilitates the identification of patients who may derive greater benefit from intensive TKI/mTOR-targeted regimens while potentially requiring alternative combination strategies to overcome the immunometabolic barriers and metabolic vulnerabilities of the NMA-driven “proliferative engine”.

### 4.5 Limitations and future directions

Our multi-modal validation across 53 clinical cases and spatial metabolomics provides robust *in situ* evidence. Nevertheless, future studies employing longitudinal lineage tracing or functional MT knockouts in organoid models are needed to further clarify the precise causal drivers behind niche reconstruction. Additionally, exploring the synergy between MT inhibitors and immunotherapies in the context of “immune-active” early cores could open new therapeutic avenues.

## 5 Conclusion

This study characterized NMA as a hallmark of ccRCC progression and spatial niche reconstruction (**Figure 7**), offering a novel, clinically actionable framework for metabolic risk stratification via MT-CO1.

## Data Availability

Publicly available datasets were analyzed in this study. The multi-omics data for the TJ-RCC cohort analyzed in this study are publicly available external datasets, which were deposited by the original investigators on Zenodo at https://zenodo.org/record/8063124. The public single-cell RNA sequencing data used for analyzing the paired normal, primary tumor, and metastatic lesions were obtained from the Gene Expression Omnibus (GEO) under accession number GSE202813. Large-scale transcriptomic and clinical data for the TCGA-KIRC cohort were acquired from the GDC Data Portal (https://portal.gdc.cancer.gov/). The clinical and pathological data of the independent validation cohort (53 cases) from Meizhou People’s Hospital are not publicly available due to privacy and ethical restrictions regarding human subjects. However, these data, along with the custom scripts used for spatial neighborhood analysis and NMA quantification, are available from the corresponding author upon reasonable request.
