# Supplementary material for: Nucleo-mitochondrial asymmetry profiles the proliferative engine and spatial niche reconstruction in clear cell renal cell carcinoma
Source: Front Immunol. 2026 Jun 12;17:1838182. doi: 10.3389/fimmu.2026.1838182 (PMC13306239; doi:10.3389/fimmu.2026.1838182)
Supplement: Supplementary file 1 [file Table1.docx]

**Supplementary Figure 1**


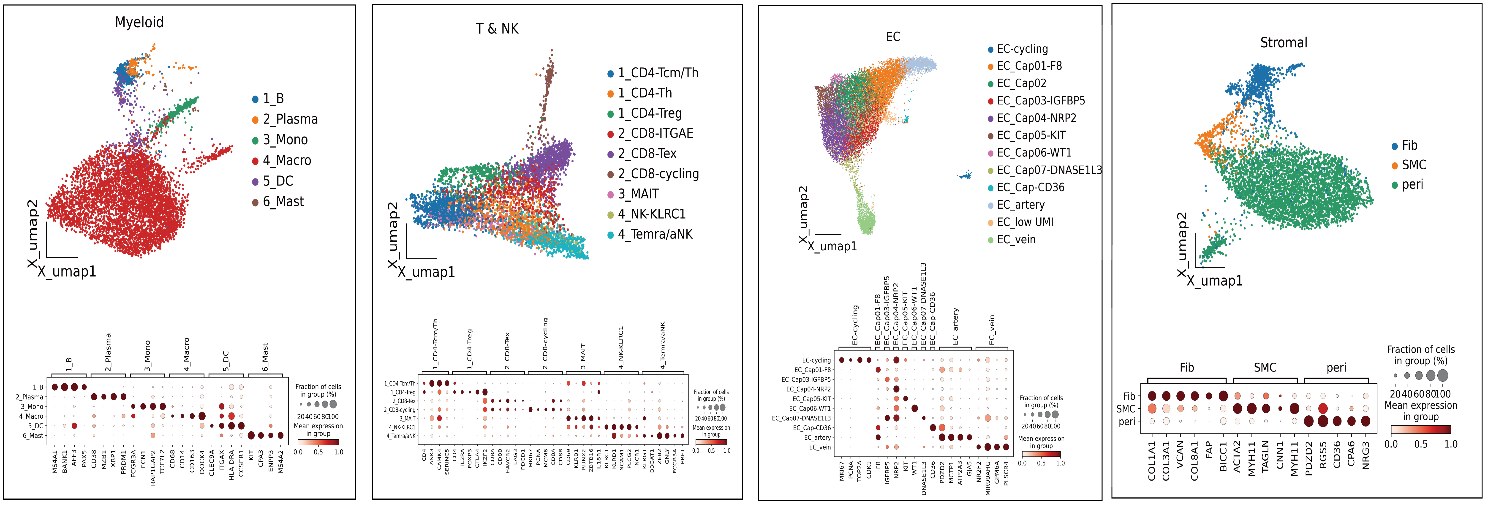


**Supplementary Figure 1: Further sub-clustering and marker genes for myeloid, T&NK, EC, and stromal cell types.2. ccRCC subpopulations have two phenotypes: proliferation and invasiveness**

**Supplementary Figure 2**

**
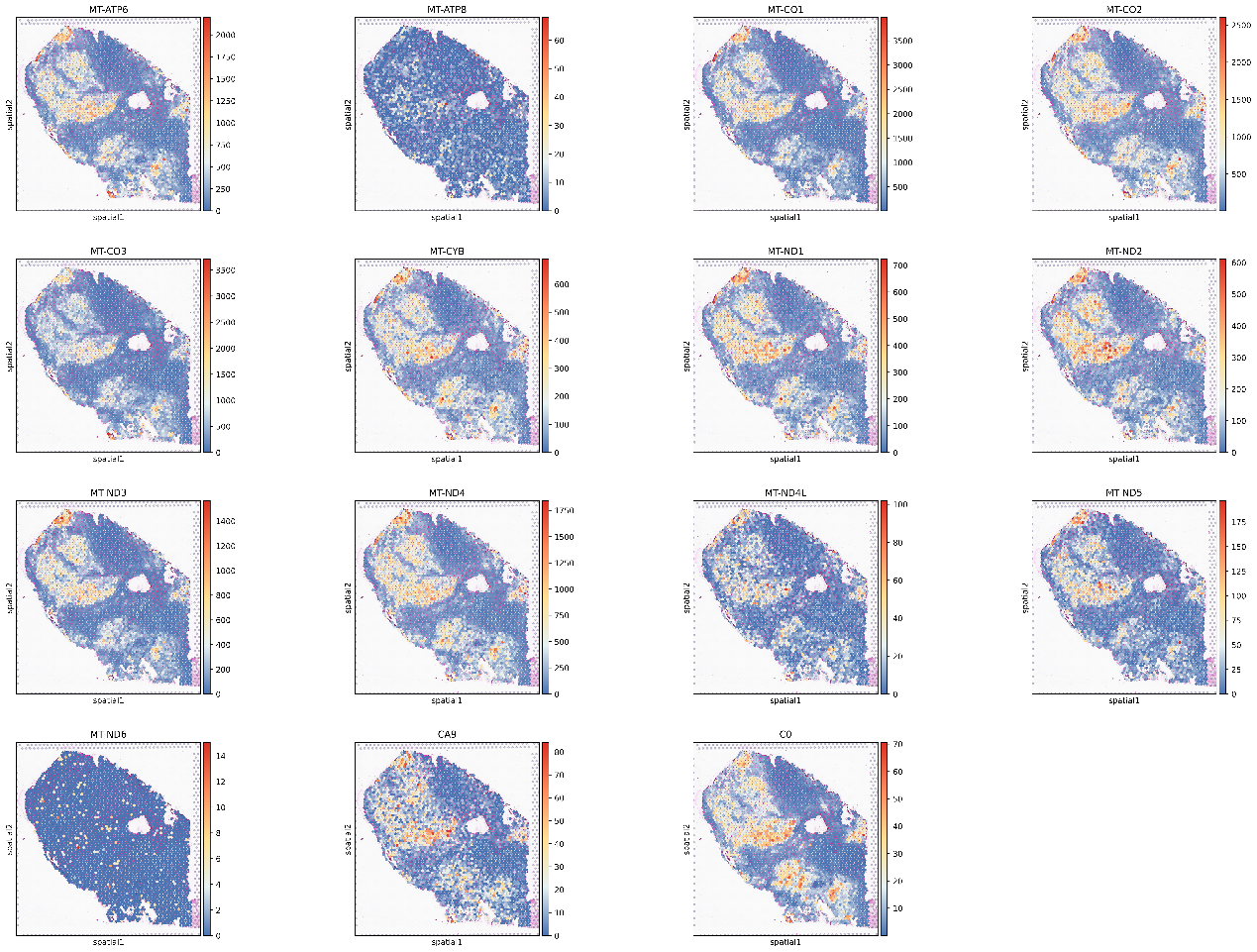
**

**S2:** The spatial distribution of all MT genes was consistent with that of C0 subpopulation, except MT-ND6 and MT-ATP8 which showed scattered distribution.

**Supplementary Figure 3**

**
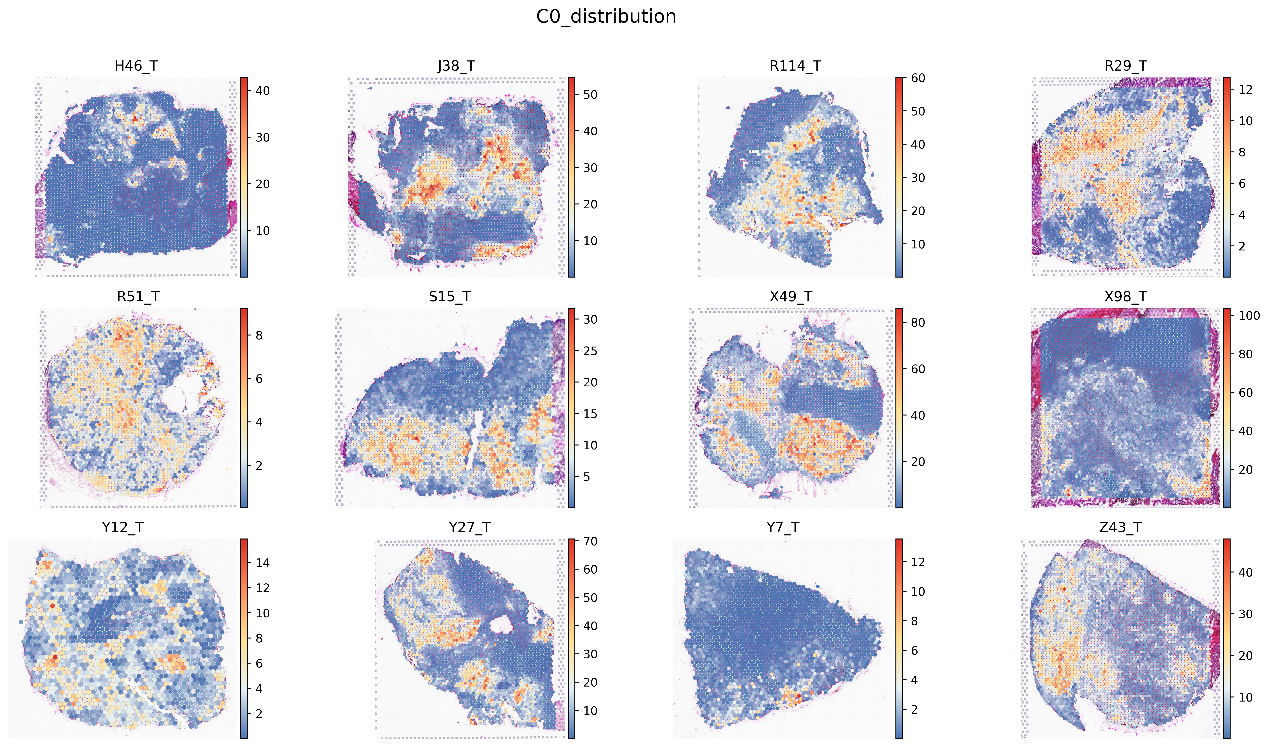
**

**
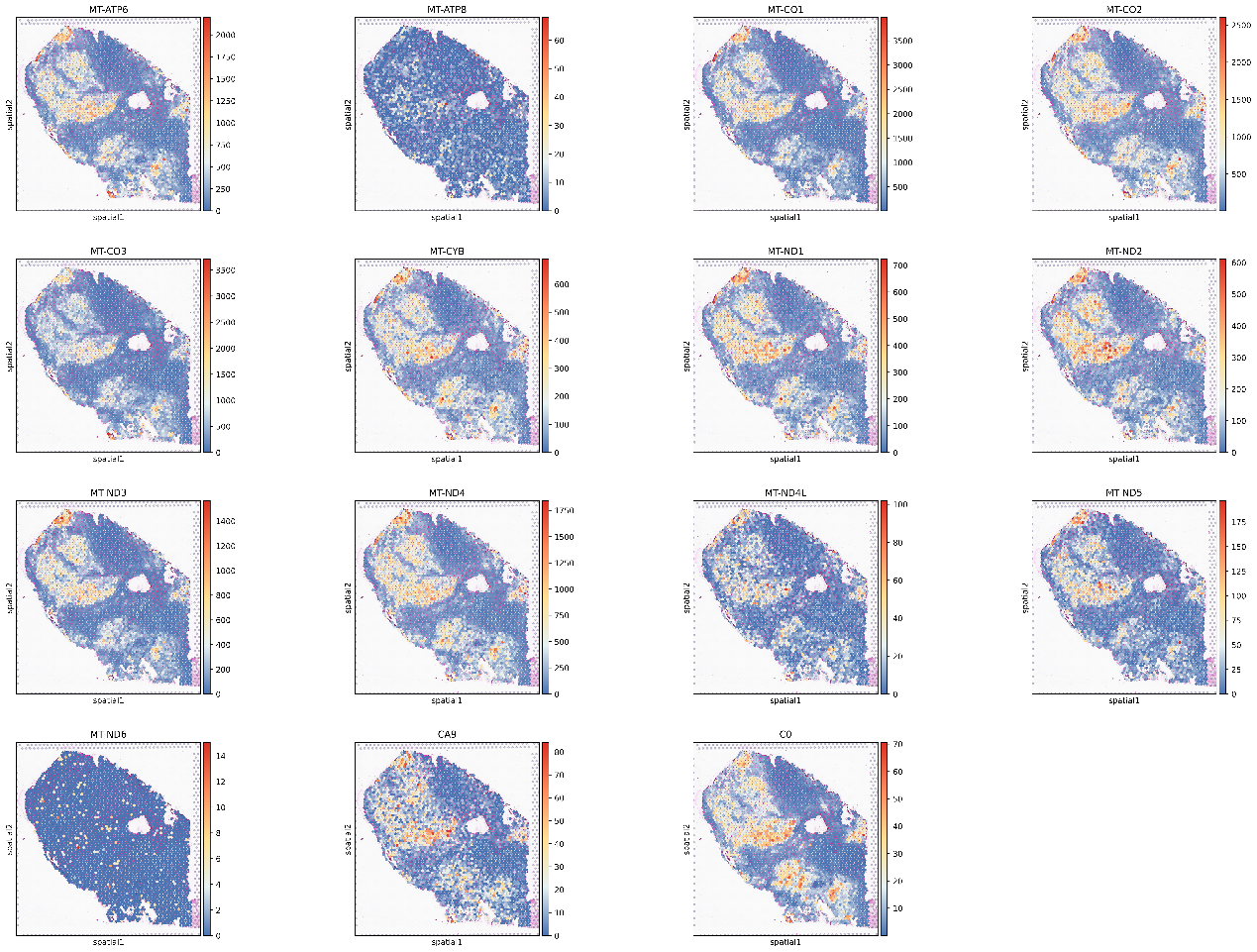

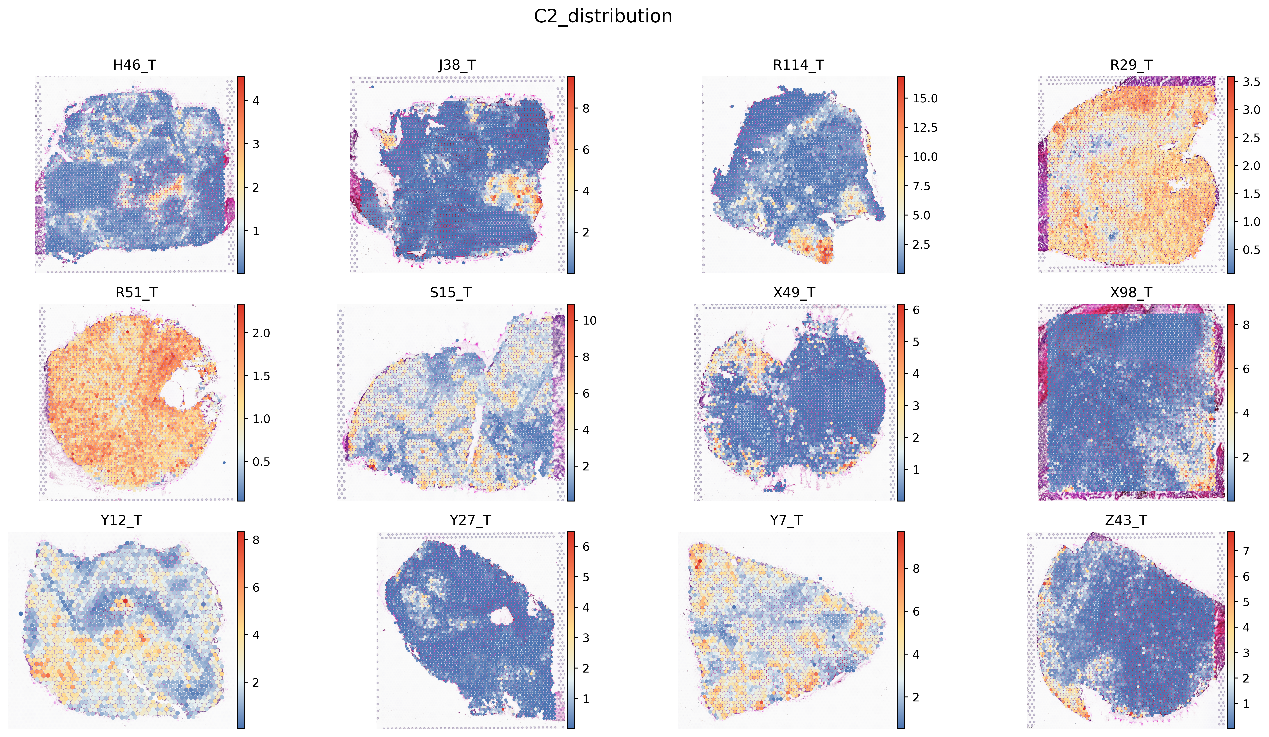
**

**S3:** Spatial distribution of the three ccRCC subpopulations of CO,C1, and C2 in 12 samples
